# Revision of *Cyrtandra* (Gesneriaceae) in the Marquesas Islands

**DOI:** 10.3897/phytokeys.30.6147

**Published:** 2013-11-27

**Authors:** Warren L. Wagner, Anthony J. Wagner, David H. Lorence

**Affiliations:** 1 Department of Botany, MRC-166, National Museum of Natural History, Smithsonian Institution, P.O. Box 37012, Washington, DC 20013-7012; 2 National Tropical Botanical Garden, 3530 Papalina Road, Kalaheo, HI 96741-9599, USA

**Keywords:** *Cyrtandra*, Gesneriaceae, Marquesas Islands, French Polynesia, conservation

## Abstract

During the preparation of the *Vascular Flora of the Marquesas Islands* three new species of *Cyrtandra* (Gesneriaceae) have come to light and are described herein: *C. uapouensis* W. L. Wagner & Lorence, *C. uahukaensis* W. L. Wagner & Lorence, and *C. kenwoodii* W. L. Wagner & A. J. Wagner. Amended descriptions of the eight previously described Marquesan species are also provided as well as a key to the species. With the description of these the new species *Cyrtandra* in the Marquesas Islands consists of 11 species, six of which have been included in recent molecular phylogenetic studies of Pacific *Cyrtandra*, and appear to have arisen from one original introduction. If the other five species are members of this Marquesas clade then *Cyrtandra* would represent the largest lineage of Marquesas vascular plants. *Psychotria* is largest genus in the Marquesas Islands with 13 species, but is thought to consist of three separate lineages.

## Introduction

*Cyrtandra* J.R. Forster & G.Forster (Gesneriaceae) is a genus of about 650 species with a paleotropical distribution from Southeast Asia and throughout the Pacific islands (Atkins et al. in press). *Cyrtandra* represents approximately 15–20% of the species within the family Gesneriaceae, and is a major component of the Old World members of the family, which are members of subfamily Cyrtandroideae Endlicher (Burtt and Wiehler 1995). The greatest concentration of species is in the Malesian region where 450 to 600 species occur (Atkins et al. in press). *Cyrtandra* species form a conspicuous component of the understory in forests across this vast region. Although the genus is readily dispersed, occurring on virtually all archipelagoes across the Pacific islands to the remote Hawaiian Islands, most species are local endemics, with distributions on one island or occasionally on several within a single archipelago. Recent phylogenetic studies of *Cyrtandra* (Burtt 2001; Cronk et al. 2005; Clark et al. 2008, 2009) support a Southeast Asian origin of the genus, with subsequent dispersal and diversification throughout the Pacific. These studies identify a single strongly supported Pacific oceanic island clade that dates back ±22 million years (Clark et al. 2009).

Brown (1935) described the first species of *Cyrtandra* from the Marquesas Islands in his Gesneriaceae treatment for the *Flora of Southeastern Polynesia*. In the treatment he recognized four species (*Cyrtandra feaniana*, *Cyrtandra ootensis*, *Cyrtandra nukuhivensis*, and *Cyrtandra toviana*), all currently recognized here, but both *Cyrtandra ootensis* and *Cyrtandra nukuhivensis* with modified delimitations. Brown also described a fifth species, *Cyrtandra jonesii*,but placed it in a new genus, *Cyrtandroidea*, which he erroneously believed to be a member of the Campanulaceae. He mentioned the stamens and style as features that place this plant with otherwise clear features of a *Cyrtandra* in the Campanulaceae. Gillett (1973) provided an updated revision of the Marquesas species as part of a reconsideration of the south Pacific species. He recognized the same five species as Brown and made the combination for *Cyrtandroideajonesii* to bring it properly into the genus as *Cytrandra jonesii*. Fosberg and Sachet (1981) in their revision of Marquesan *Cyrtandra* maintained all five species. They also added three more species revealed by then recent collecting (*Cyrtandra tahuatensis*, *Cyrtandra revoluta*) and by a new interpretation of collections from Nuku Hiva considered by Gillett to represent *Cyrtandra jonesii* (now *Cyrtandra thibaultii*). Collecting in the Marquesas Islands intensified greatly with the initiation of the current *Vascular Flora of the Marquesas Islands* project under the direction of Warren L. Wagner and David H. Lorence (Lorence and Wagner 2011; Wagner and Lorence 1997) resulting in discovery of two additional species (*Cyrtandra uahukaensis*, *Cyrtandra kenwoodii*) and a reinterpretation of *Cyrtandra nukuhivensis* to exclude the Ua Pou populations as *Cyrtandra uapouensis*, bringing the total to 11 species in the Marquesas Islands (Table 1).

**Table 1.** Island distribution of Marquesas species of *Cyrtandra*. Islands arranged from oldest to youngest.

Species / Island	Nuku Hiva	Ua Huka	Ua Pou	Hiva Oa	Tahuata	Fatu Hiva
*Cyrtandra jonesii*	X?	X				
*Cyrtandra nukuhivensis*	X					
*Cyrtandra thibaultii*	X		X			
*Cyrtandra uahukaensis*		X				
*Cyrtandra uapouensis*			X			
*Cyrtandra kenwoodii*			X			
*Cyrtandra feaniana*	X	X		X	X	
*Cyrtandra ootensis*		X		X	X	X
*Cyrtandra tahuatensis*				X	X	X
*Cyrtandra revoluta*						X
*Cyrtandra toviana*	X					
**Totals**	4	4	3	3	3	3

**Figure 1.**
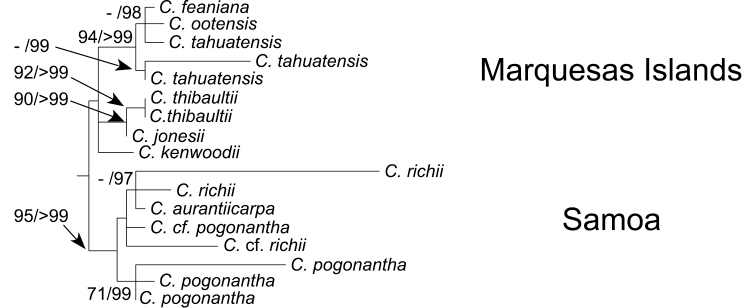
A portion of the Maximum likelihood phylogram from Clark et al. (2009) based on analysis of ITS, ETS and psbA-trnH regions. Numbers along branches indicate branch support (bootstrap support/Bayesian posterior probabilities).

**Figure 2.**
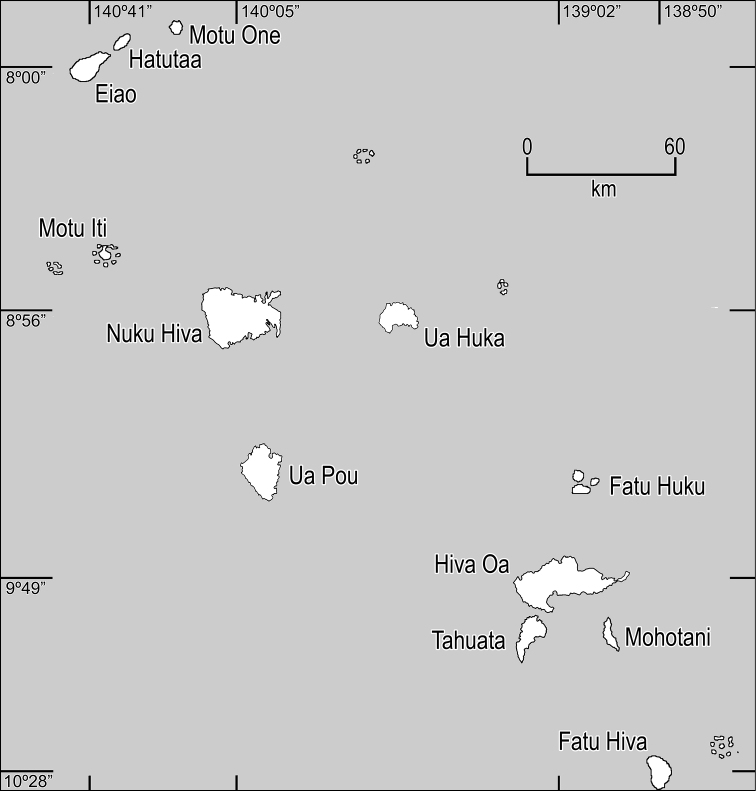
Map of the Marquesas Islands.

The Marquesas Island species form a weakly supported clade in the recent maximum likelihood molecular analyses (Clark et al. 2009) with six of the 11 species sampled. Figure 1 presents a portion of the overall tree with the Marquesas species and the sister group to them from Clark et al. (2009). The Marquesas species are divided into two major strongly supported clades with one outlier, *Cyrtandra kenwoodii* from Ua Pou, that is unplaced in a polytomy with the other two clades. The two primary clades in the molecular analysis are corroborated by morphology: species with calyx divided nearly to the base forming one clade (with all three species sampled, *Cyrtandra feaniana*, *Cyrtandra ootensis*, and *Cyrtandra tahuatensis*), and the species with plicate calyx, which are divided less than 3/4 of their length forming the second clade (with only *Cyrtandra jonesii* and *Cyrtandra thibaultii* sampled). Within the divided calyx clade there are three samples of *Cyrtandra tahuatensis*. They do not form a clade but rather the two samples from Tahuata form a clade sister to a polytomy of the Fatu Hiva sample and the single samples of the other two species. Several of the unique Marquesas species were not included, especially the peltate-leaved *Cyrtandra toviana* and the revolute-leaved *Cyrtandra revoluta*, in the analyses so that a conclusion of a single introduction for all of the species cannot be substantiated until there has been further sampling and perhaps more variable sequence data added since support for some parts of the clade are not robust (Clark et al. 2009). The plicate calyx clade occurs on the older islands of the Northern Marquesas group (Nuku Hiva, Ua Huka, and Ua Pou) while the divided calyx clade occurs primarily on the younger islands of the southern group (Hiva Oa, Tahuata, and Fatu Hiva) (see Fig. 2 for Map of the the Marquesas Islands). There are a few collections of the divided calyx clade from the older islands, all of which were collected by the Pacific Entomological Survey in 1929–1930. If the locality information on the labels of these collections is correct, the species of the divided calyx clade appear to no longer occur on the older islands.

When evaluated using the IUCN Red list categories and criteria for threatened species version 3.1 (IUCN 2001, see also http://www.iucnredlist.org/static/categories_criteria_3_1), all 11 of the Marquesan species of *Cyrtandra* fall into the Endangered (EN) or Critically Endangered (CR) categories, which designate species facing the highest risk of extinction in the wild. Marquesan species of *Cyrtandra* meet the IUCN criteria by having known ranges less than 100 km^2^, an area of occupancy of less than 10 km^2^, and continuing decline in the quality of habitat across the Marquesas Islands (Florence and Lorence 1997; Mueller-Dombois and Fosberg 1998; Meyer and Salvat 2009).

All measurements given herein are taken from dried herbarium specimens, although certain features such as shapes were supplemented with information from alcohol-preserved flowers and fruits, field notes, and color slides or digital photos. Measurements are presented in the descriptions as follows: length × width, followed by units of measurement (mm or cm). Specimens from the following herbaria were studied: AD, BISH, BKL, CBG, CHR, E, HAST, K, L, MO, MPU, NSW, NY, P, PAP, PTBG, S, UC, US, and WU). Many of the Marquesas *Cyrtandra* species are very narrowly distributed so distribution maps would not be especially useful so we provide here (Fig. 2) a map of the Marquesas Islands to show the islands and their relationship to one another.

## Taxomic part

### Artifical key to species of Marquesas Cyrtandra

**Table:** 

1	Leaves suborbicular, peltate	*Cyrtandra toviana*
–	Leaves elliptic, elliptic-oblanceolate or lanceolate, not peltate	2
2	Leaves strongly revolute	*Cyrtandra revoluta*
–	Leaves flat or very inconspicuously revolute	3
3	Plant less than 1 m tall; leaves 1–2.1 cm wide	*Cyrtandra kenwoodii*
–	Plant (1–) 2–5 m tall; leaves (1.3–) 5–25 cm wide	4
4	Calyx divided less than 3/4 the way to the base, plicate	5
–	Calyx divided nearly to the base, not plicate	9
5	Leaves rugose, 26–40 × 10–25 cm; bracts rhombic, 18–20 mm	*Cyrtandra jonesii*
–	Leaves not rugose, 14–29 × 5–13 cm; bracts triangular, 3–11 mm	6
6	Inflorescence 14–30 cm; peduncles 80–90 mm; calyx 25–45 mm	*Cyrtandra thibaultii*
–	Inflorescence 0–7 cm; peduncles 0–35 mm; calyx 15–25 mm	7
7	Lower surface of leaves densely dark ferruginous pubescent	*Cyrtandra uahukaensis*
–	Lower surface of leaves pale ferruginous pubescent when young, glabrate or pubescent primarily along veins on lower surface at maturity	8
8	Leaves elliptic, occasionally elliptic-oblanceolate, 14–24.5 × 5–7.5 cm; calyx 15–23 mm	*Cyrtandra nukuhivensis*
–	Leaves elliptic-oblanceolate to elliptic, 19–28 × 8–13 cm; calyx 20–25 mm	*Cyrtandra uapouensis*
9	Plants glabrous, or occasionally with a few scattered hairs	*Cyrtandra feaniana*
–	Plants pubescent, at least on the youngest parts of the plant	10
10	Plant densely ferruginous pubescent; peduncles 2–3 mm in diameter	*Cyrtandra tahuatensis*
–	Plant sparsely to moderately whitish to ferruginous pubescent; peduncles 1–2 mm in diameter	*Cyrtandra ootensis*

### Plicate calyx group

#### 1. Cyrtandra jonesii

(F. Br.) G. W. Gillett, Univ. Calif. Publ. Bot. 66: 55. 1973.

http://species-id.net/wiki/Cyrtandra_jonesii

Fig. 3

##### Basionym.

*Cyrtandroidea jonesii* F. Br., Bernice P. Bishop Mus. Bull. 130: 324. 1935.

##### Type.

Marquesas Islands: Ua Huka: Hanay [Hane] Bay, 500 m, 16 November 1922, W. B. Jones 1712 (lectotype: BISH-501566!; designated by G. W. Gillett, Univ. Calif. Pub. Bot. 66: 55. 1973). H. St. John made a superfluous lectotypification (Phytologia 33: 422, 1976). A second syntype, Quayle 1243 (BISH) [now referred to *Cyrtandra thibaultii*] was mentioned as a “second type sheet” by Brown.

##### Description.

Shrubs 2–6 m. Leaves opposite, borne on upper 2–5 nodes, leathery when fresh, drying chartaceous, broadly elliptic to broadly elliptic-oblanceolate, 26–40 × 10–25 cm, rugose, upper surface glossy, green, sparsely pubescent when young, especially along major veins, quickly glabrate, lower surface pale green, moderately ferruginous appressed pubescent, especially along major veins, quickly glabrate, margins irregularly serrate-dentate, apex broadly rounded, ± with an often long, acuminate tip, base broadly rounded, truncate or abruptly attenuate, petioles 2.5–7.0 cm long. Flowers in loose, open cymes 8–20 cm long, basally cauliflorouson the main stem, cymules 1–3 flowered, peduncles up to 30 mm long, ca. 3–4 mm in diameter, pedicels 0–1 mm, bracts rhombic, whitish brown, 18–20 mm, soon deciduous; calyx funnelform, whitish brown, plicate, 33–46 mm long, the lobes 10–20 mm long, unequal, triangular, sparsely minutely appressed pubescent, appearing glabrate, deciduous after anthesis; corolla white, tube funnelform, 40–50 mm long, slightly exceeding the calyx, lobes suborbicular, 8–15 mm long; style ca. 7 mm long, glabrous or with a few scattered hairs. Mature berry unknown, but young ones ovoid, ca. 10 mm long.

##### Distribution.

Marquesas Islands, endemic to Ua Huka, scattered in Hitikau area to Vaikivi summit and drainage, from 500 to 870 m. One collection (Mumford & Adamson 561, BISH) made in 1929 is from Nuku Hiva, but it is possible that the locality on the label is incorrect and the plant was actually collected on Ua Huka, where Adamson was for four days in October 1929 (N. Evenhuis, pers. comm. May 2013).

##### Ecology.

*Cyrtandra jonesii* is known only in montane wet shrubland with *Freycinetia impavida* (Gaudich. ex Hombr.) B. C. Stoneand *Hibiscus tiliaceus* L. dominant.

##### Conservation status.

IUCN Red List Category: Endangered EN B1ab (i,ii,iii) + 2 ab (i,ii,iii): B2: total area of occupancy less than 5000 km^2^ (ca. 423 km^2^). B1a, severely fragmented; B1b (i–iii), habitat continuing decline inferred. The suitable habitat for *Cyrtandra jonesii* on Ua Huka (ca. 83 km^2^) and possibly on Nuku Hiva (ca. 340 km^2^), is restricted to mountain slopes and summits, indicated as an endangered environment that is threatened by human activity (deforestation and fire), feral animals, and invasive plants, reducing the extent of the forest (Florence and Lorence 1997; Mueller-Dombois and Fosberg 1998; Meyer and Salvat 2009). If this species is restricted to only Ua Huka, then its conservation status would be CR.

##### Specimens examined.

**Marquesas Islands. Nuku Hiva:** Puokohe, 3500 ft [1067 m], 22 October 1929, Mumford 561 (BISH, S). **Ua Huka:** Hitikau region, ascended via the Matukuoha Ridge over-looking Hane, constitutes the summit of the single crater of Ua Huka, 730 m, UTM 0661697 – 9015668, 5 Dec 2003, Wood 10493 (PTBG), 780 m, Wood 10474, (PTBG), 700 m, Wood 10484 (PTBG); summit of Hitikau area, 872 m, 8°54'30.6"S, 139°31'46.9"W, 15 June 2004, Perlman & Wood 19017 (PTBG, US); Hitikau summit area, 686 m, 8°54'19.7"S, 139°31'7.3"W, 26 July 2005, Perlman & Meyer 19741 (BISH, P, PAP, PTBG, US); Hitikau and the Vaikivi summit region, 870 m, 8°54'40.5"S, 139°31'32.5"W, 14-15 June 2004, Wood & Perlman 10744 (PAP, PTBG, US); Vaikivi summit region and drainage, boulder-strewn stream-bed running south and west below Hitikau, 700 m, 8°54'S, 139°31'W, 14–15 June 2004, Wood & Perlman 10751 (PTBG).

##### Discussion.

Brown described the genus *Cyrtandroidea* for this species in the Campanulaceae, but Gillett (1973) effectively placed the generic name in synonymy when he transferred it to *Cyrtandra*. Gillett, however, did not recognize that the second syntype, Quayle 1243 (BISH) represented another species, subsequently described by Fosberg and Sachet (1981) as *Cyrtandra thibaultii*. Fosberg and Sachet pointed out that Gillett’s description of *Cyrtandra jonesii* was based primarily on his own collection of the then undescribed *Cyrtandra thibaultii*. Here we have revised the description, based on new collections and on the description from original material provided by Fosberg and Sachet (1981).

**Figure 3.**
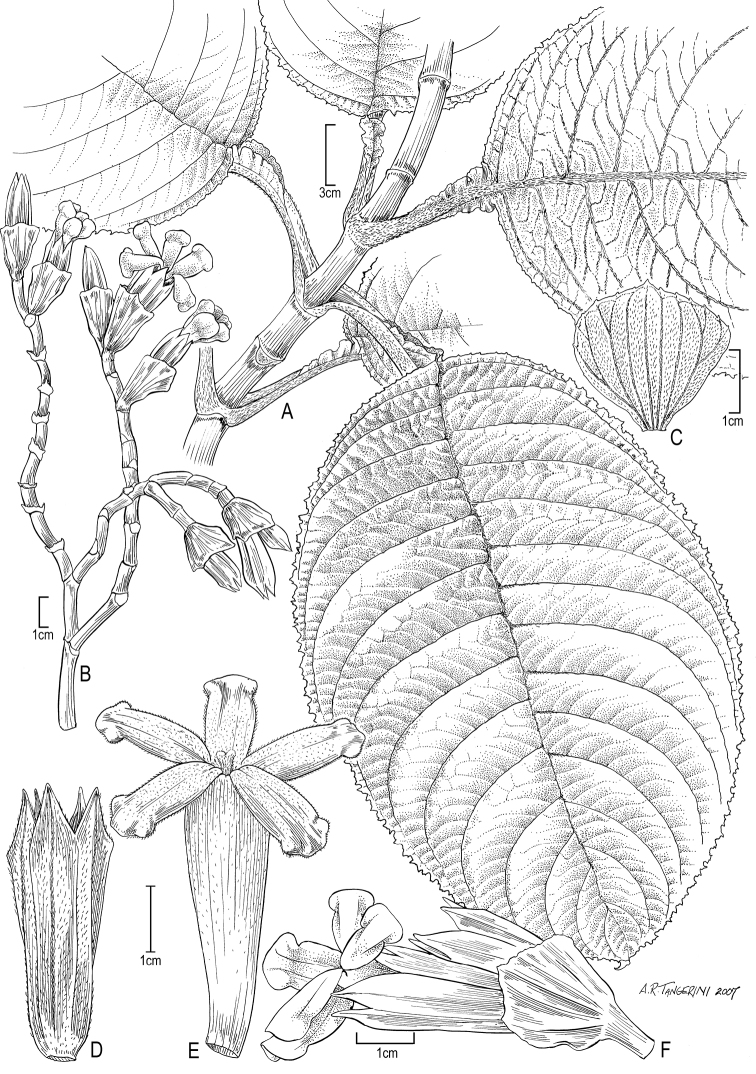
*Cyrtandra jonesii* (F. Br.) G. W. Gillett **A** Habit **B** Inflorescence **C** Bract **D** Calyx **E** Corolla **F** Flower, lateral view. Drawn from Wood 10744 (PTBG) and field photographs.

#### 2. Cyrtandra nukuhivensis

F. Br., Bernice P. Bishop Mus. Bull. 130: 274. 1935.

http://species-id.net/wiki/Cyrtandra_nukuhivensis

Fig. 4

##### Type.

Marquesas Islands: Nuku Hiva: Rain forest, 800 m, September 1922, E.H. Quayle 1233 [1293] (holotype: BISH-509954!; isotypes: BISH!, BKL).

##### Description.

Shrubs (1.5–) 2–5 m tall; stems usually few. Leaves opposite, borne on upper 4–8 nodes, elliptic, occasionally elliptic-oblanceolate, 14–24.5 × 5–7.5 cm, glabrate, upper surface glossy, dark green, lower surface pale green, margins serrulate, apex rounded and usually with acuminate tip, base attenuate, petioles 3–6 cm. Flowers in congested cymes 2.0–7 cm arising in the upper leaf axils, flowers 1–9, ferruginous pubescent, quickly glabrate, peduncles 10–35 mm long, ca. 1–2 mm in diameter, pedicels 2–18 mm, bracts triangular, ca. 3–5 mm; calyx ellipsoid, white, plicate, 15–23 mm long, the lobes 6–11 mm long, deciduous after anthesis; corolla broadly funnelform, tube ca. 22–30 mm, slightly exceeding the calyx, the lobes ca. 8–10 mm long; style ca. 3 mm long, pubescent. Young berry cylindrical-ovoid, 10–18 mm long.

##### Distribution.

Marquesas Islands, uncommon, endemic to Nuku Hiva, Toovii Plateau to Mt. Ooumu, from 800 to 1150 m.

##### Ecology.

*Cyrtandra nukuhivensis* is known from montane wet forest with *Metrosideros collina* (J. R. Forst. & G. Forst.) A. Gray and *Weinmannia marquesana* F. Br. forest with diverse fern understory along with other shrubs and trees such as species of *Cheirodendron*, *Coprosma*, *Crossostylis*, *Ilex*, *Melicope*, and *Xylosma*.

##### Conservation status.

IUCN Red List Category: Endangered EN B1ab (i,ii,iii) + 2 ab (i,ii,iii): B2: total area of occupancy less than 5000 km^2^ (ca. 340 km^2^). B1a, severely fragmented; B1b (i–iii), habitat continuing decline inferred. The suitable habitat for *Cyrtandra nukuhivensis* on Nuku Hiva (ca. 340 km^2^), is restricted to mountain slopes and summits, indicated as an endangered environment that is threatened by human activity (deforestation and fire), feral animals, and invasive plants, reducing the extent of the forest (Florence and Lorence 1997; Mueller-Dombois and Fosberg 1998; Meyer and Salvat 2009).

##### Specimens examined.

**Marquesas Islands. Nuku Hiva:** Toovii, 800 m, Quayle 1293 (BISH [2]); Toovii, flanc N de l’épaulement SE du Mt. Ooumu, 960 m, 29 May 1984, Florence 6851 (BISH, P); Toovii, épaulement S du Mt. Ooumu, 930 m, 8°51'S, 140°8'W, 8 December 1982, Florence 4337 (BISH [2], K, NY, P, US); Toovii, flanc SE du Mt. Ooumu, 1020 m, 8°50'S, 140°09'W, 11 March 1986, Florence 7523 (BISH, US); Toovii Plateau, spur of Mt. Ooumu, 950 m, 18 July 1977, Gagné 1104 (BISH) Gagné 930 m, 1105 (BISH, US); Toovii Plateau, l’Economie Rurale, summit, above new road, on crest, 1152 m, 16 July 1988, Perlman, Wagner, Lorence & Florence 10100 (BISH, E, MO, PAP, PTBG, US); ravine en forêt de Toovii, 2 March 1973, Hallé 2073 (US); between Taiohae Bay and Hooumi Bay, 900 m, 20 July 1977, Gagné & Montgomery 1160 (BISH); S slope of Mt. Tapuaooa, 10 July 1970, Gillett 2178 (BISH, US); Mt. Ooumu, 3500 ft, 28 March 1960, Decker 376 (BISH, US [2]); Toovii, Ooumu area, top of Tapueahu Valley off new hwy, 1067–1128 m, 8°51'S, 140°19'W, 20–22 September 1995, Wood & Perlman 4626, (PAP, PTBG, US, WU); Ooumu area, top of Tapueahu Valley off new Hwy, 1067–1128 m, 8°51'53"S, 140°10'6.3"W, 23 June 1997, Wood, Meyer, Luce & Tetuanui 6340 (PTBG); summit of ridge S of Tekao, 0.5 mile N New Airport Road, between Airport Road & Tekao, main ridge above Toovii, 1128 m, 25 September 1995, Perlman & Wood 15060 (BISH, P, PAP, PTBG, US, WU); along new Airport road, along summit crest above Toovii, Peak #1227 M., summit of Mts. S of Tekao, 1122 m, 22 September 1995, Perlman & Wood 15034 (BISH, MO, P, PAP, PTBG [2], US, WU).

##### Discussion.

*Cyrtandra nukuhivensis* is delimited in a more restricted way than previously and is here considered to be endemic to Nuku Hiva. The Ua Pou plants are here considered a distinct, but closely related species.

**Figure 4.**
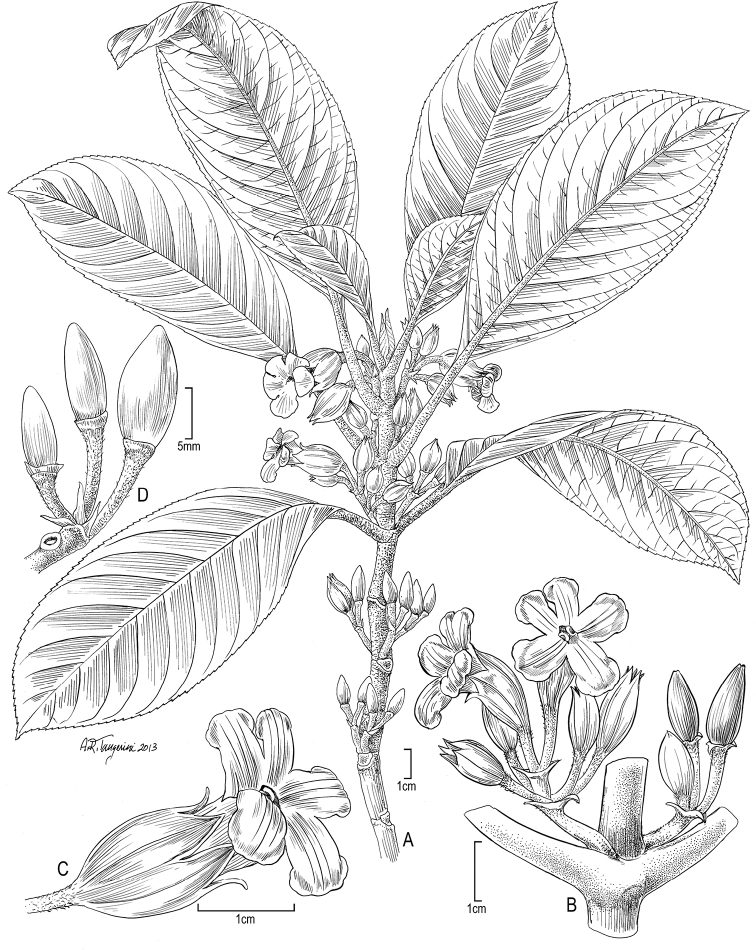
*Cyrtandra nukuhivensis* F. Br. **A** Habit **B** Inflorescence **C** Flower, lateral view **D** Fruit. Drawn from field images and Perlman 15060 (US, PTBG), except A from Perlman 15034 (US).

#### 3. Cyrtandra thibaultii

Fosberg & Sachet, Smithsonian Contr. Bot. 47: 28. 1981.

http://species-id.net/wiki/Cyrtandra_thibaultii

Fig. 5

##### Type.

Marquesas Islands: Nuku Hiva: Tovii, 850–900 m, 9 July 1975, J.-C.Thibault 134 (holotype: US-02969235!; isotype: US!).

##### Description.

Shrubs 2–5 m. Leaves opposite, borne on upper 4–8 nodes, oblong-elliptic to elliptic, 16–29 × 6.5–13 cm, upper surface glabrate or ferruginous pubescent on veins, lower surface densely or moderately ferruginous pubescent, apex acuminate, base cuneate, petioles 2–6 cm long, appressed ferruginous pubescent. Flowers in open, loose cymes, 14–30 cm long, arising in the upper leaf axils, appressed ferruginous pubescent, peduncle 80–90 mm long, ca. 1–2 mm in diameter, pedicels 10–15 mm, elongating to 25–30 mm in fruit, bracts triangular, ca. 6–11 mm; calyx cylindrical-ellipsoid, pale green, plicate, 25–45 mm long, appressed ferruginous pubescent, lobes 5–17 mm long, deciduous after anthesis; corolla funnelform, tube 25–50 mm long, lobes about 15–20 mm long; style 10 mm long, appressed to spreading pubescent. Berry fusiform, ca. 45 mm long. Seeds broadly elliptic or ovoid, 0.3-0.4 mm long, reticulate.

##### Distribution.

Marquesas Islands, scattered, on Nuku Hiva from the Toovii Plateau to Mt. Ooumu, and on Ua Pou from Oave, Teavahaakiti, and Pouakei, from 700 to 1050 m.

##### Ecology.

*Cyrtandra thibaultii* is known from *Metrosideros collina*, *Weinmannia marquesana*, and *Freycinetia impavida* forest with an association with diverse shrubs and trees such as species of *Cheirodendron*, *Coprosma*, *Crossostylis*, *Hernandia*, *Ilex*, *Melicope*, *Psychotria*, *Trimenia*, and *Xylosma*.

##### Conservation Status.

IUCN Red List Category: Endangered EN B1ab (i,ii,iii) + 2 ab (i,ii,iii). B2: total area of occupancy less than 5000 km^2^ (ca. 445 km^2^). B1a, severely fragmented; B1b (i–iii), habitat continuing decline inferred. The suitable habitat for *Cyrtandrathibaultii* on Nuku Hiva (ca. 340 km^2^) and Ua Pou (ca. 105 km^2^), is restricted to mountain slopes and summits, indicated as an endangered environment that is threatened by human activity (deforestation and fire), feral animals, and invasive plants, reducing the extent of the forest (Florence and Lorence 1997; Mueller-Dombois and Fosberg 1998; Meyer and Salvat 2009).

##### Specimens Examined.

**Marquesas Islands. Nuku Hiva:** Toovii Valley, 3 July 1970, Gillett 2156 (BISH, US); forêt de montagne à l’ouest de Toovii, 2 March 1973, Hallé 2064 (US); Toovii region, NW of l’Economie Rurale complex along new road to airport over flanking mountains, 1020–1030 m, 3 August 1988, Lorence, Wagner, Montgomery, Florence & Bishop 6268 (PAP); Toovii, épaulement S du Mt. Ooumu, 880 m, 8°51'S, 140°8'W, 1 December 1982, Florence 4232 (BISH [2], K, NY, P, US); Toovii, vallon du réservoir, 830 m, 8°52'S, 140°9'W, 26 May 1984, Florence 6737 (BISH [2]); Toovii Plateau, spur of Mt. Ooumu, 790 m, 16 July 1977, Gagné 1048 (BISH, US); W part of Toovii, along new road W of l’Economie Rurale, 1170 m, 16 July 1988, Wagner, Lorence, Florence & Perlman 6096 (BISH, P, US) & 6097 (BISH, MO, US); Toovii Plateau, l’Economie Rurale, along new road, 994 m, 16 July 1988, Perlman, Wagner, Lorence & Florence 10095 (BISH, PTBG, US); Toovii, N of agriculture station, along drainage and up to ridge, 808 m, 8°50'8.6"S, 140°8'6.8"W, 22 June 1997, Wood & Meyer 6325 (MPU, P, PAP, PTBG, US, WU), Wood & Meyer 6325-A (PTBG); between Taiohae Bay and Hooumi Bay, >700 m, 20 July 1977, Gagné 1150 (BISH, US); S of Airport road, drainages of Tapueahu gulch, to NW of Toovii over summit crest, 963 m, 21 September 1995, Perlman & Wood 15020 (BISH, MO, P, PAP, PTBG, US, WU). **Ua Pou:** Vallon en contrebas de la crête reliant Poumaka et la crête sommitale Oave-Teavahaakiti, 750 m, 22 July 2003, Meyer 2541 (BISH, P, PAP, PTBG, US); Pou Maka, ridge between Pou Maka and Oave, 792 m, 9°23'7.7"S, 140°4'W, 22 July 2005, Perlman 19730 (P, PAP, PTBG, US); forested ridge and slopes up to Pouakei, northwest side, 930 m, 9°24'S, 140°5'W, 21 Nov 2003, Wood 10428 (PTBG, US); forested ridge and slopes up to Teavahaakiti, northwest side, 914 m, 24 Nov 2003, Wood 10449 (PTBG, US).

##### Discussion.

The sample used (Wood 10428) in the molecular study by Clark et al. (2009) was identified as *Cyrtandra nukuhivensis*, but during this taxonomic revision it was re-identified as the closely related *Cyrtandra thilbaultii*.

**Figure 5.**
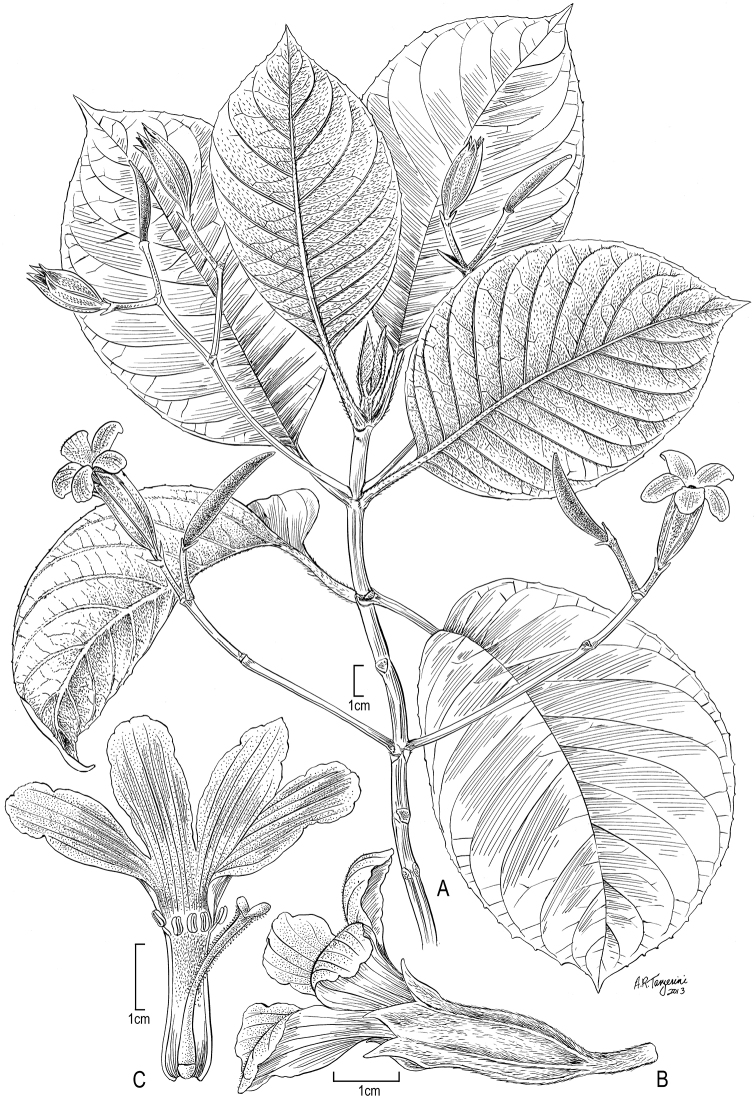
*Cyrtandra thibualtii* Fosberg & Sachet **A** Habit, S. Perlman et al 10095 (PTBG) **B** Flower, lateral view **C** Corolla, longitudinal section. Drawn from Perlman et al 10095 (US) and field images.

#### 4. Cyrtandra uahukaensis

W. L. Wagner & Lorence sp. nov.

urn:lsid:ipni.org:names:77134229-1

http://species-id.net/wiki/Cyrtandra_uahukaensis

Fig. 6

##### Type.

Marquesas Islands: Ua Huka: Summit of ridge near Hitikau, *Hibiscus tiliaceus* forest, 820 m, 28 June 1997, S. Perlman, K. Wood, & J.-Y. Meyer 15844 (holotype: PTBG-026355!; isotype: US!).

##### Description.

Shrubs 1-2 m tall; stems apparently few. Leaves opposite, borne on upper 2–5 nodes, broadly elliptic, 18–26 × 7–10 cm, lower surface densely ferruginous appressed pubescent, upper surface glabrate, irregularly dentate, the teeth variable in size, petiole 5–6.5 cm, ferruginous appressed to spreading pubescent. Flowers in cymes arising in the upper leaf axils, apparently 1–3 flowered, peduncle 0–2 mm, ca. 1–2 mm in diameter, pedicels up to 6 mm; calyx cylindrical-ellipsoid, white, plicate; corolla much longer than calyx; style up to 10 mm, flower parts otherwise unknown. Partly mature berries up to 14 mm long.

##### Distribution.

Marquesas Islands, rare, endemic to Hitikau summit area, Ua Huka, from 820–870 m.

##### Ecology.

*Cyrtandra uahukaensis* is known only from *Freycinetia impavida* - *Hibiscus tiliaceus* shrubland.

##### Etymology.

The specific epithet refers to the island of Ua Huka where the only known populations occur.

##### Conservation status.

IUCN Red List Category: Critically Endangered CR B2a + 2b (i, ii, iii). B2: total area of occupancy less than 10 km^2^ (ca. 5 km^2^). B2a, a single population known; b (i–iii), habitat continuing decline inferred. The suitable habitat for *Cyrtandra uahukaensis* on Ua Huka (ca. 83 km^2^) is indicated as an endangered environment, threatened feral animals, and invasive plants, reducing the extent of the forest (Florence and Lorence 1997; Mueller-Dombois and Fosberg 1998; Meyer and Salvat 2009).

##### Specimens examined.

**Marquesas Islands. Ua Huka:** 13 May 1918, Henry 7 (P); Hitikau summit region, large bowl-like plateau, 823–884 m, 8°54'22"S, 139°31'66"W, 28 June 1997, Wood 6380 (PTBG); Hitikau and the Vaikivi summit region, 790 m, 8°54'S, 139°31'W, 14–15 June 2004, Wood & Perlman 10756 (PTBG).

##### Discussion.

Little is known about this species as it has been collected only four times. None of the collections were preserved with any flowers, only young fruit. *Cyrtandra uahukaensis* is characterized by the densely pubescent leaves and condensed inflorescences. It grows in the same area as *Cyrtandra jonesii* and appears to be closely related. Their relationships and ecology should be studied as it seems atypical for two very closely related species of the genus to grow in near sympatry.

**Figure 6.**
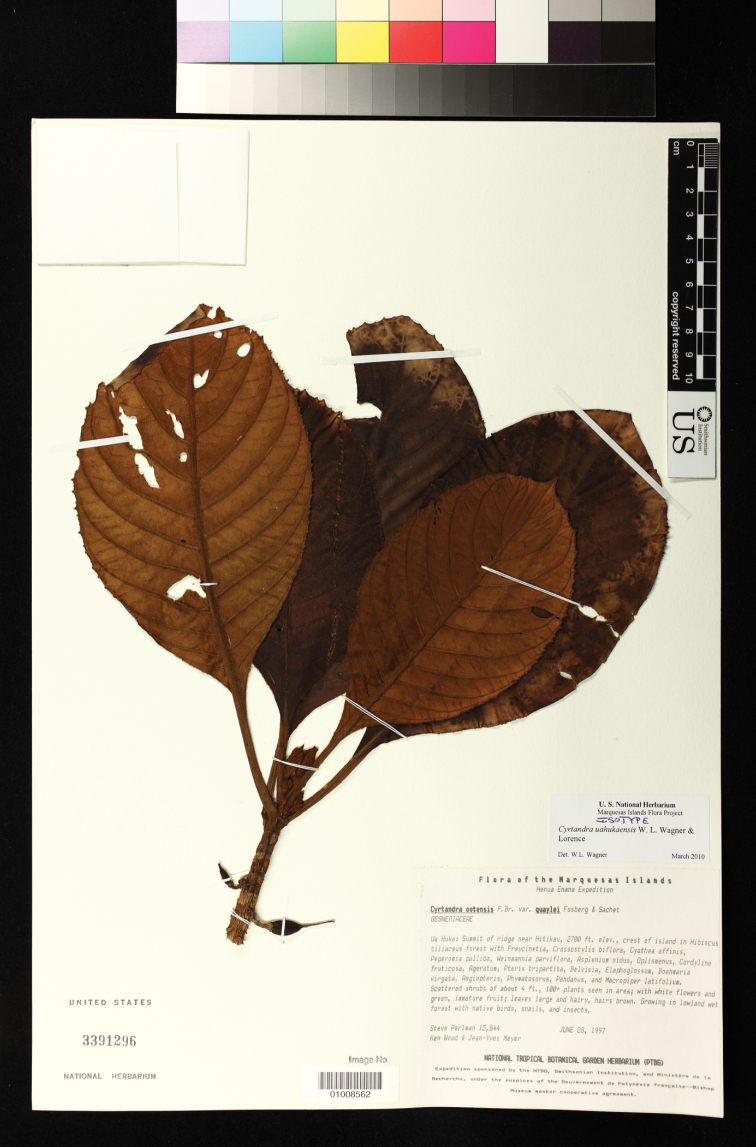
*Cyrtandra uahukaensis* W. L. Wagner & Lorence (Perlman et al. 15844, isotype US).

#### 5. Cyrtandra uapouensis

W. L. Wagner & Lorence sp. nov.

urn:lsid:ipni.org:names:77134230-1

http://species-id.net/wiki/Cyrtandra_uapouensis

Fig. 7

##### Type.

Marquesas Islands: Ua Pou: Ua Pou: Ridge just north of Oave, between Oave and Matahenua, high mountain peaks along main backbone ridge, 945 m, 9°23'455"S, 140°4'433"W, 3 July 2004, S. P. Perlman & K. R. Wood 19085 (holotype: PTBG-042428!; isotypes: BISH!, MO, NY, P, PAP, US!).

##### Description.

Shrubs 1.5–3 m tall; stems usually few. Leaves opposite, borne on upper 3–5 nodes, elliptic-oblanceolate to elliptic, 19–28 × 8–13 cm, upper surface glossy, dark green, glabrate, lower surface pale green, ferruginous pubescent when young, when maturing pubescent primarily along the veins, margins serrulate, apex rounded and usually acuminate, base attenuate, petioles 4.5–7 cm. Flowers in congested cymes 1–3 cm long arising in the upper leaf axils, flowers 1–6, ferruginous pubescent, quickly glabrate, peduncles 3–15 mm long, ca. 1–2 mm in diameter, pedicels 0–10 mm, bracts triangular, ca. 8–11 mm; calyx ellipsoid, white, plicate, 20–25 mm long, the lobes 8–13 mm long, deciduous after anthesis; corolla broadly funnelform, tube ca. 22–35 mm, slightly exceeding the calyx, the lobes ca. 8–10 mm long; style ca. 3 mm long, pubescent. Young berry ellipsoid-ovoid, 15–25 mm long.

##### Distribution.

Marquesas Islands, rare, endemic to high ridges of Ua Pou, from Oave, Matahenua, Teavahaakiti, Tekahuipu, and Tekohepu, 680–945 m.

##### Ecology.

*Cyrtandra uapouensis* is known only in *Metrosideros collina*–*Weinmannia marquesana* wet forest with diverse fern understory and other shrubs and trees such as species of *Cheirodendron*, *Coprosma*, *Crossostylis*, *Ilex*, *Melicope*, and *Xylosma*.

##### Etymology.

The specific epithet refers to the island of Ua Pou where the only known populations occur.

##### Conservation status.

IUCN Red List Category: Critically Endangered CR B2a + 2b (i, ii, iii). B2: total area of occupancy less than 10 km^2^ (ca. 5 km^2^). B2a, a single population known; b (i–iii), habitat continuing decline inferred. The suitable habitat for *Cyrtandra uapouensis* on Ua Pou (ca. 105 km^2^) is indicated as an endangered environment, threatened feral animals and invasive plants, reducing the extent of the forest (Florence and Lorence 1997; Mueller-Dombois and Fosberg 1998; Meyer and Salvat 2009).

##### Specimens examined.

**Marquesas Islands. Ua Pou:** Mt. Tekahoipu, 800 m, 9 September 1922, Quayle 1151 (BISH); crête sud menant au mont Teavahaakiti, vallon à pente forte, zone semi-ouverte, 810 m, 20 Jun 2004, Meyer 2847 (P, PAP, PTBG, US); Teavahaakiti, steep slopes of main ridge to S of Oave, N & E facing cliffs between Teavahaakiti & Tekohepu, 683 m, 5 July 1997, Perlman & Wood 15904 (MO, P, PAP, PTBG, US, WU).

##### Discussion.

Gillett (1973) included the only known specimen of *Cyrtandra uapouensis* at that time (*Quayle 1151*) within his delimitation of *Cyrtandra nukuhivensis*; populations from Ua Pou are here separated based on its occurrence on a different island and its consistently larger leaves. *Cyrtandra uapouensis* grows sympatrically with *Cyrtandra kenwoodii*, and there are several collections (cited in hybrid section) that are morphologically intermediate between them that appear to represent hybrids. The fact that *Cyrtandra kenwoodii* forms a separate branch in the phylogenetic analyses (Clark et al. 2009; fig. 1) from both the divided calyx group and the plicate calyx group suggests that perhaps this hybridization has impacted the genome of *Cyrtandra kenwoodii* or that the species is of hybrid origin.

**Figure 7.**
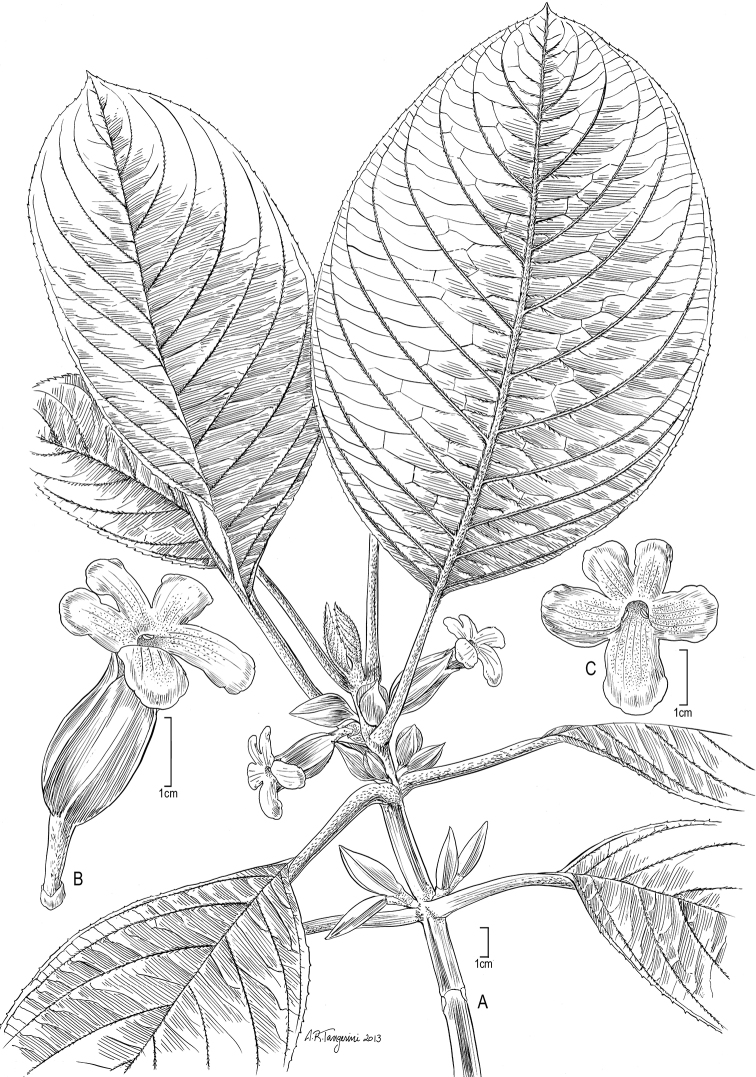
*Cyrtandra uapouensis* W. L. Wagner & Lorence **A** Habit **B** Flower, lateral view **C** Flower, face view. Drawn from Perlman 19085 (isotype, US) [**A, C**] and Perlman 15904 (PTBG) [**A, B**] and field images.

#### 6. Cyrtandra kenwoodii

W. L. Wagner & A. J. Wagner sp. nov.

urn:lsid:ipni.org:names:77134231-1

http://species-id.net/wiki/Cyrtandra_kenwoodii

Fig. 8

##### Type.

Marquesas Islands:  Ua Pou: Tekohepu, windswept and cloud-shrouded summit, shrubland of *Metrosideros collina* with *Dicranopteris linearis*, *Paesia rugulosa*, *Freycinetia*, *Blechum*, *Oleandra sibbaldii*, and *Selliguea feeioides*, 2500-3000 ft [762–914 m], 4–5 July 1997, K. R. Wood & S. Perlman 6467 (holotype: US-3390815!; isotype: PTBG!).

##### Description.

Shrubs 0.2–1 m. Leaves opposite, clustered on upper 2–5 nodes, brittle, elliptic to elliptic-oblanceolate, 4–7.6 × 1.0–2.1 cm, upper surface glossy, green, glabrous, lower surface pale green, glabrous, margins irregularly and inconspicuously serrulate, apex acuminate, base attenuate, petioles 0.2–1.6 cm long. Flowers in loose, open cymes 3–6 cm long, arising in the upper leaf axils, cymes 1–3 flowered, peduncles 15–40 mm long, ca. 1–1.5 mm in diameter, pedicels 10–18 mm, bracts lanceolate, ca. 6–8 mm; calyx funnelform, white, ca. 11–15 mm long, lobes 5–9 mm long, subequal, glabrous, deciduous after anthesis; corolla white, funnelform, tube ca. 24–26 mm long, lobes ca. 6–8 mm long. Immature berry 15 mm long.

##### Distribution.

Marquesas Islands, rare, endemic to high ridges of Ua Pou, from Oave and Matahenua to Teavahaakiti, Tekahuipu, and Tekohepu, 790–945 m.

##### Ecology.

*Cyrtandra kenwoodii* is known only in cloud-swept summits with *Metrosideros collina* shrubland with other shurbs and small trees such as *Apetahia*, *Freycinetia*, and *Hibiscus*.

##### Etymology.

This new species is named for Kenneth R. Wood, who first collected it and who has contributed greatly to our knowledge of the flora of the Marquesas and the Hawaiian Islands through his collections and field observations.

##### Conservation status.

IUCN Red List Category: Critically Endangered EN B2a + B2b(i–iii). B2: total area of occupancy less than 10 km^2^ (ca. 5 km^2^). B2a, a single population known; b (i–iii), habitat continuing decline inferred. The suitable habitat for *Cyrtandra kenwoodii* on Ua Pou (ca. 105 km^2^) is indicated as an endangered environment, threatened feral animals and invasive plants, reducing the extent of the forest (Florence and Lorence 1997; Mueller-Dombois and Fosberg 1998; Meyer and Salvat 2009).

##### Specimens examined.

**Marquesas Islands. Ua Pou:** Central Ua Pou including the summit crest regions around Oave and the near-by peak of Matahenua, 899-924 m, 9°23'45.4"S, 140°4'43.3"W, 2 July 2004, Wood & Perlman 10804 (PTBG, US), 10813 (PTBG); ridge just north of Oave, between Oave and Matahenua, high mountain peaks along main backbone ridge, 945 m, 9°23'45.5"S, 140°4'43.3"W, 3 July 2004, Perlman & Wood 19088 (PTBG, US); Teavahaakiti, steep slopes of main ridge to S of Oave, N & E facing cliffs between Teavahaakiti & Tekohepu, 869 m, 5 July 1997, Perlman & Wood 15911 (MO, P, PAP, PTBG, US, WU).

##### Discussion.

The sample of *Cyrtandra kenwoodii* used (*Wood & Perlman 10804*) in the molecular study by Clark et al. (2009) was identified as *Cyrtandra feaniana*, prior to realization that the diminutive plants from Ua Pou represented an undescribed species. The molecular data show that *Cyrtandra kenwoodii* is in a polytomy along with the two primary clades of Marquesan species, but is grouped here with the plicate species as it has the calyx only divided about half way to the base, but is not plicate as far as is known.

**Figure 8.**
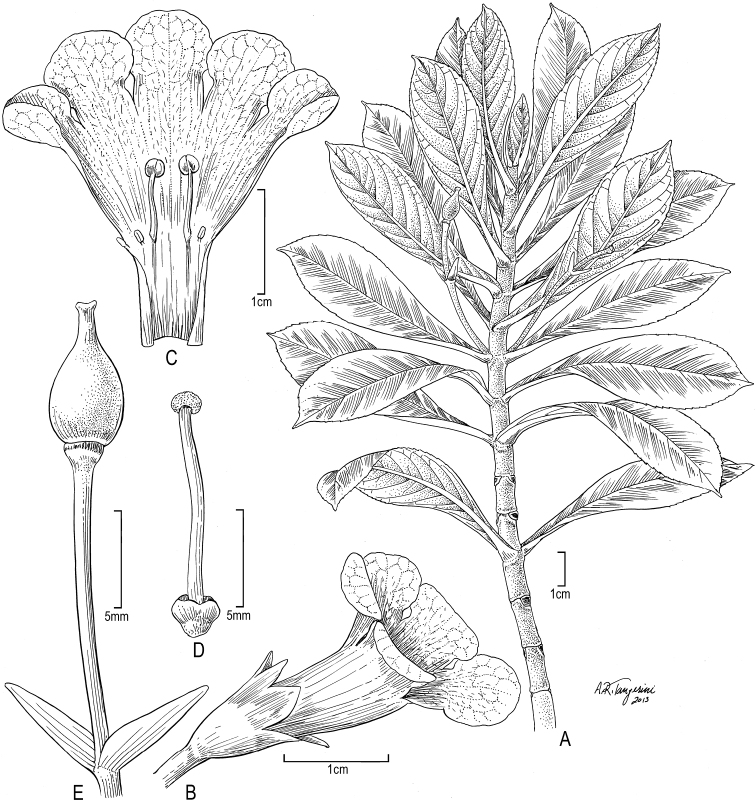
*Cyrtandra kenwoodii* W. L. Wagner & A. J. Wagner **A** Habit **B** Flower, lateral view **C** Corolla, longitudinal view **D** Pistil. Drawn from Wood 10804 (US), except **A** from Wood 6467 (holotype, US).

### Divided calyx group

#### 7. Cyrtandra feaniana

F. Br., Bernice P. Bishop Mus. Bull. 130: 272. 1935.

http://species-id.net/wiki/Cyrtandra_feaniana

Fig. 9

##### Type.

Marquesas Islands: Hiva Oa: Feani, 800 m, 8 December 1921, F.B.H. Brown 827 (holotype: BISH-509530!)

##### Description.

Shrub 1.5–3 m; stems glabrous or with a few scattered hairs when young. Leaves opposite, lanceolate to elliptic, 2.5–19 × 1.3–7.2 cm, glabrous, margins crenulate-serrulate, apex acute or acuminate, base cuneate, petioles 1–5 cm. Flowers 1-3 in cymes arising in the leaf axils, usually somewhat shorter than the leaves, glabrous or with scattered hairs, peduncles 10–45 mm long, ca. 0.5–1.5 mm in diameter, pedicels 18–35 mm long, bracts inconspicuous, narrowly lanceolate or triangular, 1–5 mm long, deciduous; calyx usually white, occasionally greenish white, 10–24 mm, divided nearly to the base, lobes lanceolate, tardily deciduous; corolla white, glabrous externally, tube 18–23 mm long, the lobes subequal, broadly obovate, 10–12 mm long; ovary narrowly ovoid, glabrous, style ca. 7 mm long, minutely puberulent. Berries pale orange, ca. 15 mm long, narrowly ovoid, glabrous.

##### Distribution.

Marquesas Islands, occurring on Hiva Oa and Tahuata, and known from a few collections made in 1922 on Nuku Hiva and Ua Huka, from 700 to 1100 m.

##### Ecology.

*Cyrtandra feaniana* is known from ridges and summit areas of montane wet forest dominated by *Metrosideros collina* and other shrubs and trees such as *Weinmannia marquesana* forest with diverse fern understory and other shrubs and trees such as species of *Coprosma*, *Crossostylis*, *Freycinetia*, *Ilex*, *Melicope*, *Polyscias*, *Psychotria*, and *Xylosma*.

##### Conservation status.

IUCN Red List Category: Endangered EN B1ab (i,ii,iii) + 2ab (i,ii,iii). B2: total area of occupancy less than 5000 km^2^ (ca. 799 km^2^). B1a, severely fragmented; B1b (i–iii), habitat continuing decline inferred. The suitable habitat for *Cyrtandra feaniana* on Nuku Hiva (ca. 340 km^2^), Ua Huka (ca. 83 km^2^), Hiva Oa (ca. 315 km^2^), and Tahuata (ca. 61 km^2^) is restricted to mountain slopes and summits, indicated as an endangered environment that is threatened by human activity (deforestation and fire), feral animals, and invasive plants, reducing the extent of the forest (Florence and Lorence 1997; Mueller-Dombois and Fosberg 1998; Meyer and Salvat 2009).

##### Specimens examined.

**Marquesas Islands. Nuku Hiva:** Toovii, 800–1000 m, October 1922, Quayle 1335 (BISH, BKL, US). **Ua Huka:** 9 November 1922, Quayle, 1792 (BISH); 9 November 1922, Quayle 1750 (BISH). **Hiva Oa:** Feani, 800 m, Brown 827 (BISH); Feani, 3900 ft, 23 January 1932, LeBronnec 804 (BISH); Feani ridge to upper slopes of dry side of island, 1050 m, 12 February 1975, Oliver & Schäfer 3152 (BISH, CBG, US); Feani, 1000 m, 11 November 1989, MacKee & Cherrier 44704 (BISH); sentier d’Atuona à la crête de Feani, haute vallée côté Atuona, 980 m, 6 November 1975, Schäfer 5943 (BISH, CBG, CHR, NSW, PTBG, US [2]); Atuona–Feani Trail, crest of ridge and top of leeward slope, 1200–1300 m, 24-26 September 1963, Sachet & Decker 1152 (BISH, CBG, CHR, MO, NSW, PTBG, US [2]); Mt. Feani, trail from Atuona to Hanamenu, 1180 m, 10 February 1975, Oliver & Schäfer 3101 (BISH, CBG, CHR, L, MO, NSW, PTBG,US); Mt. Feani, trail from Atuona to Hanamenu, 1180 m, 11 February 1975, Oliver & Schäfer 3111 (BISH, US); Mt. Feani, trail from Atuona to Hanamenu, 1120 m, 5 March 1975, Oliver & Schäfer 3238 (BISH, CBG, CHR, NSW, US), Oliver & Schäfer 3239 (BISH, CBG, US); 30 m above camp near “the source” (Vaiumete) on trail from Atuona to Hanamenu, 1000 m, 29 Jan 2003, Price, Dunn & Lorence 200 (P, PAP, PTBG, US); Feani area, on Hanamenu trail, summit crest, heading from Vaiumete et Vaiumioi (source) toward Hanamenu, 1090 m, 9°47'9.86"S, 139°4'7.06"W, 30 Jan 2003, Perlman, Wood, Lorence, Meyer & Dunn 18348 (BISH, P, PAP, PTBG, US); Atuona, piste de Hanamenu, NW du Mt. Temetiu, 1090 m, 9°48'S, 139°5'W, 30 July 1988, Florence, Lorence, Perlman & Wagner 9634 (BISH, K, P, PAP, PTBG, US); chemin d’Atuona à Hanamenu par Feani, 1040 m, 11 February 1975, Schäfer 5174 (MPU), Schäfer 5174B (MPU), Schäfer 5174C (MPU); Vaipahee Falls area, ridge crest, Kaava ridge further toward Feani, 914 m, 9 August 1988, Perlman 10261 (AD, BISH, E, F, MO, NY, P, PTBG, US); trail to Feani and Hanamenu, 3300 ft [1006 m], 29 July 1988, Perlman, Wagner, Lorence & Florence 10178 (BISH); trail to Feani and Hanamenu, along plateau rim and ridge trail, 1097 m, 30 July 1988, Perlman 10184 (BISH, PAP, PTBG); trail to Hanamenu, 1000 m, 9°47'9.29"S, 139°4'56.7"W, 1 August 2005, Perlman 19760 (AD, BISH, NY, P, PAP, PTBG, US); Matauuna, 27 February 1930, Pacific Entomol. Surv. HO 1004 b (BISH); above Atuona, 700 m, 6 October 1930, Pacific Entomol. Surv. Ex 47 (BISH); 3900 ft [1189 m], 23 January 1932, Pacific Entomol. Surv. 6B 804 (BISH); windswept summit, along trail between Mt. Feani and Timetiu, 1100 m, 30 July 1988, Wagner & Lorence 6223 (BISH, P, PTBG, US); N side of Mt. Temetiu, 1100 m, 23 March 1929, Mumford & Adamson 151 (BISH, S, UC); NE slope of Mt. Temetiu, 2200 ft, 24 July 1929, Mumford & Adamson 467 (BISH, S); N side of Mt. Temetiu, 1100 m, 9 October 1930, Pacific Entomol. Surv. 151 (BISH); Temetiu, 1189 m, 9°48'S, 139°4'W, 25 August 1995, Wood 4378 (BISH, MO, PAP, PTBG, US); summit of Temetiu, top of highest peak, 1262 m, 25, August 1995, Perlman, Wood & Meyer 14880 (PAP, PTBG, US, WU); summit of Mt. Ootua, 920 m, 10 May 1929, Mumford & Adamson 388 (BISH, S, UC); Mt. Ootua, central part, 860 m, 29 July 1977, Gagné 1214 (BISH, US); Mt. Ootua, off road between Airport and Puamau, along ridge and summit, on N facing slope, 841- 866 m, 21 August 1995, Perlman & Wood 14863 (AD, BISH, MO, P, PAP, PTBG, US, WU); Mt. Ootua, summit area, 830 m, 9°46'25"S, 138°58'27.5"W, 19 Feb 2003, Perlman 18476 (BISH, P, PAP, PTBG, US); Mt. Ootua summit area, 838 m, 9°45'9.90"S, 138°58'29.5"W, 19 July 2004, Perlman & Wood 19215 (BISH, P, PAP, PTBG, US). **Tahuata:** Summit of ridge above Vaitahu, near Haaoiputeomo, on ridge near antenna, along ridge crest between Vaitahu & Hanatetena, 823 m, 1 September 1995, Perlman, Wood & Luce 14923 (PAP, PTBG, US); summit ridge near Haaiputeomo, satellite dish region NE of Vaitahu, 762–823 m, 9°57'19"S, 139°5'7.4"W, 17–19 July 1997, Wood 6570 (BISH, P, PAP, PTBG, US, WU); Haaoiputeomo, on SE side of slope above village of Hanatetena, summit ridge of island, 793 m, 2 September 1995, Perlman, Wood & Luce 14932 (BISH, P, PAP, PTBG, US, WU); ridge between Amatea & Haaoiputeomo, SE facing slopes and cliffs over Hanatetena village, 835 m, 11 July 1997, Perlman, Wood & Luce 15953 (PTBG, US) & 847 m, 15956 (P, PAP, PTBG, US, WU); ridge between Amatea and Haaoiputeomo, S facing slope, 786 m, 9°56'S, 139°4'W, 19 July 1997, Perlman 16025 (PAP, PTBG, US), 750 m, Perlman 16017 (MO, P, PAP, PTBG, US, WU); au-dessus de Hamatea, sur la crête centrale de U’ua’o, 850 m, 31 May 1975, Thibault 82 (BISH, CBG, PTBG, US).

##### Discussion.

*Cyrtandra feaniana* along with the closely related *Cyrtandra ootensis* are the most commonly occurring species of *Cyrtandra* in the Marquesas Islands. In addition *Cyrtandra feaniana* has the widest distribution, along with *Cyrtandra ootensis*, occurring on four islands, although seemingly rare on Nuku Hiva and Ua Huka and has not been collected on either island since 1922. Specimens with more than a few hairs on various parts of the plant are here identified as *Cyrtandra ootensis*. This includes all of the specimens from Fatu Hiva that are nearly glabrous, but have much larger leaves like other populations of *Cyrtandra ootensis*. Alternatively, it is possible that these glabrate Fatu Hiva populations represent hybrids or hybrid derivatives between *Cyrtandra feaniana* and *Cyrtandra ootensis*. This hypothesized hydridization if correct would require the colonization of Fatu Hiva by *Cyrtandra feaniana* followed by hybridization with *Cyrtandra ootensis* to produce the glabrate larger-leaved plants.

**Figure 9.**
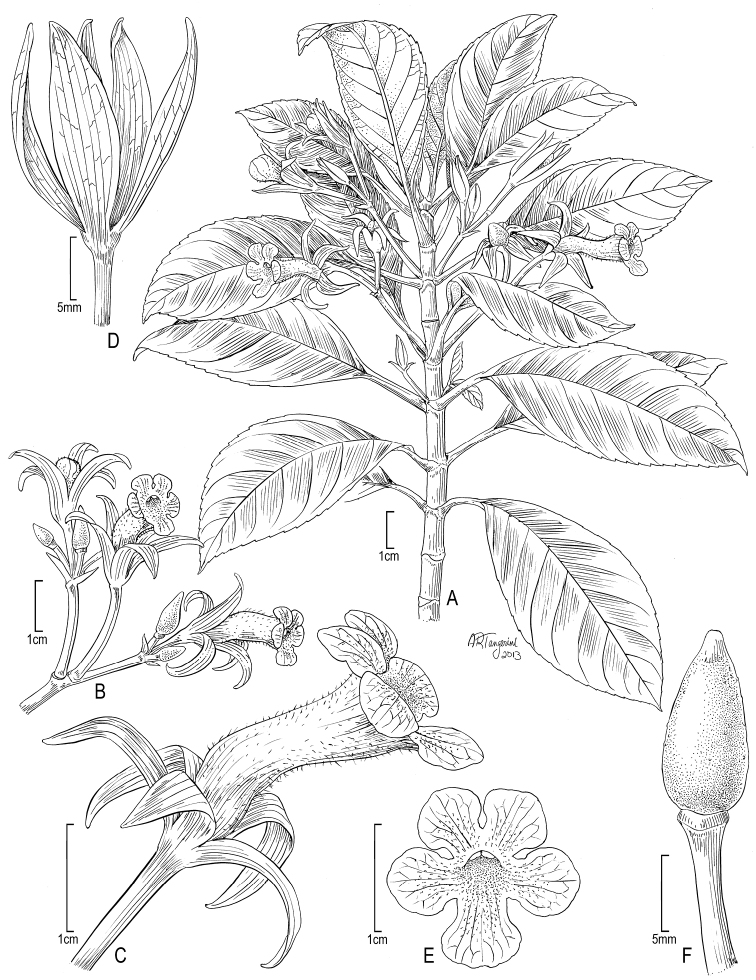
*Cyrtandra feaniana* F. Br. **A** Habit **B** Inflorescence **C** Flower, lateral view **D** Calyx **E** Flower, face view **F** Fruit. Drawn from Perlman 18476 (US) and photographs of Perlman 14932 (**A, F**), Perlman 14863 (US) and Photographs (**B, C, E**), and Schafer 980 (**D**).

#### 8. Cyrtandra ootensis

F. Br., Bernice P. Bishop Mus. Bull. 130: 273. 1935.

http://species-id.net/wiki/Cyrtandra_ootensis

Fig. 10

Cyrtandra ootensis F. Br. var. *fatuhivensis* Fosberg & Sachet, Smithsonian Contr. Bot. 47: 30. 1981. **Type.** Marquesas Islands: Fatu Hiva: Teavapuhiau Pass (above Ouia Valley), under , *Crossostylis*, *Metrosideros*, 720 m, 1–3 August 1977, B. H. Gagné 1244 (holotype: US-02969231!; isotypes: BISH [4]!).
Cyrtandra ootensis F. Br. var. *mollissima* Fosberg & Sachet, Smithsonian Contr. Bot. 47: 30. 1981. **Type.** Marquesas Islands: Hiva. Oa: Montagnes NW du Temetiu, entre la haute vallée de Hanamenu et la crête de Temetiu-Feani, 960 m, 23 October 1975, P. A. Schäfer 5923 (holotype: US-2969230!; isotypes: BISH!, P).
Cyrtandra ootensis F. Br. var. *quaylei* Fosberg & Sachet, Smithsonian Contr. Bot. 47: 31. 1981. **Type.** Marquesas Islands: Ua Huka: 600 m, 9 November 1922, E. H. Quayle 1755 (holotype: BISH-145694!).

##### Type.

Marquesas Islands: Hiva Oa: Ootua, 800 m, 15 December 1921, F.B.H. Brown 961 (holotype: BISH-509956!).

##### Description.

Shrub 1.5–4 m. Leaves opposite, elliptic to ovate, 12–28 × 5-15 cm, whitish to ferruginous pubescent, or rarely nearly glabrous, and then youngest leaves with at least some hairs, margin crenate-serrate, base unequal and broadly cuneate to attenuate, apex obtuse to acute or acuminate, petioles (1.5–) 4–10cm. Flowers 1–3 in cymes arising in the leaf axils, peduncles 5-90 mm long, ca. 1–2 mm in diameter, pedicels subtending a central pedicel 2–50 mm long, bracts lanceolate, 2–10 mm long; calyx pale green or occasionally white, 11–20 mm long, divided nearly to base, deciduous, usually densely pubescent externally; corolla tube 25–32 mm long, lobes 5–6 mm long; ovary 7–15 mm long_,_ pubescent, style 6–18 mm long, pubescent. Berries pubescent, cylindrical, 2.1 cm long. Seeds ovoid, 0.4-0.5 mm long, the coats sculptured with coarse polygonal reticulations.

##### Distribution.

Marquesas Islands, occurring on Hiva Oa and Tahuata, and two collections from Ua Huka, 670–1130 m.

##### Ecology.

*Cyrtandra ootensis* is known from ridges and summit areas of montane wet forest dominated by *Metrosideros collina* and other shrubs and trees such as species of *Coprosma*, *Crossostylis*, *Freycinetia*, *Ilex*, *Melicope*, *Polyscias*, *Psychotria*, *Weinmannia*,and *Xylosma*.

##### Conservation Status.

IUCN Red List Category: Endangered EN B1ab (i,ii,iii) + 2ab (i,ii,iii). B2: total area of occupancy less than 5000 km^2^ (ca. 564 km^2^). B1a, severely fragmented; B1b (i–iii), habitat continuing decline inferred. The suitable habitat for *Cyrtandra ootensis* on Ua Huka (ca. 83 km^2^), Ua Pou (ca. 105 km^2^), Hiva Oa (ca. 315 km^2^), and Tahuata (ca. 61 km^2^) is restricted to mountain slopes and summits, indicated as an endangered environment that is threatened by human activity (deforestation and fire), feral animals, and invasive plants, reducing the extent of the forest (Florence and Lorence 1997; Mueller-Dombois and Fosberg 1998; Meyer and Salvat 2009).

##### Specimens examined.

**Marquesas Islands. Ua Huka**: 9 November 1922, Quayle 1790 (BISH). **Hiva Oa:** Mt. Feani, trail from Atuona to Hanamenu, 1120 m, 4 March 1975, Oliver & Schäfer 3237 (BISH, CBG, US); Hanamenu region, up Hanamenu Valley to the drainages below and west of Temetiu, 884 m, 9°76'S, 139°W, 25 June 2003, Wood 10235 (PTBG); Hanamenu Valley off Hanamenu trail, 908 m, 9°47'49.6"S, 139°5'35"W, 2 August 2005, Perlman 19768 (BISH, NY, P, PAP, PTBG, US); trail toward Hanamenu, 884 m, 3 August 1988, Perlman 10207 (BISH, P, PAP, PTBG[2], US); Temetiu region, drainages to southeast of Vaimete et Vaiumioi (source), headwaters of Hanamenu, 1067 m, 30 Jan 2003, Wood 10047 (PTBG); Temetiu, 1128 m, 9°49'S, 139°4'W, 25 August 1995, Wood 4404 (BISH, P, PAP, PTBG, US); N side of Mt. Temetiu, 1100 m, 23 March 1929, Pacific Entomol. Surv. 141, (BISH, UC); Teakatau, Valley on N side of Hanamenu Trail heading down to Hanamenu past summit crest, valley between Teakatau and Tepuna, 927 m, 26 August 1995, Perlman & Meyer 14896 (PAP, PTBG, US), 933 m, 14893 (PAP, PTBG, US, WU); Atuona, 1000 m, 8 December 1921, Brown 828 (BISH); Atuona, 1100 m, 9 October 1930, Pacific Entomol. Surv. Ex 141 (BISH); chemin d’Atuona à Hanamenu par Feani, hautes pentes, côté Atuona, 935 m, 12 February 1975, Schäfer 5213 (MPU, US); Ootua, 800 m, Brown 961 (BISH); Mt. Ootua, central part, 650 m, 27 July 1977, Gagné 1176 (BISH), 900 m, 29 July 1977, Gagné 1213 (BISH); Mt. Ootua, off road between Airport and Puamau, on NW side of summit, 853 m, 21 August 1995, Perlman & Wood 14865 (PTBG, US, WU); Mts. of Vaipikopiko, new road from Hanaiapa, cut off to Vaipahee Falls, on Kaava Ridge, Vaipikopiko side, 890 m, 8 August 1988, Perlman 10232 (BISH, MO, P, PAP, PTBG, US). **Tahuata:** Haaoiputeoma, near satelite dish, NE from Vaitahu to summit ridge, 610–762 m, 1–2 September 1995, Wood 4456 (BISH, P, PAP, PTBG, US); summit ridge near Haaiputeomo, satellite dish region NE of Vaitahu, 762–823 m, 9°57'19"S, 139°5'74"W, 17– 19 July 1997, Wood 6571 (PTBG, US), 6578 (BISH, PAP, PTBG, US); ridge between Amatea and Haaoiputeomo, S facing slope, below old antenna site, 768 m, 9°56'S, 139°4'W, 19 July 1997, Perlman 16018 (AD, BISH, MO, P, PAP, PTBG, US, WU), 741 m, 16016 (BISH, P, PAP, PTBG, US). **Fatu Hiva:** ‘Omo’a Valley, 800 m, 20 January 1922, Brown 935 (BISH); slopes of Mounanui above Vaieenui Falls, on ridge top, below Maunanui, 847 m, 26 July 1988, Perlman & Florence 10171 (BISH, PTBG), 10172 (BISH, PTBG); slopes of Mounanui, 719 m, 10°28'6.63"S, 138°38'18"W, 16 July 2005, Perlman 19668 (BISH, P, PAP, PTBG, US); on ridges and gulches W side of Mounanui, 2400 ft, 10 September 1995, Perlman 14978 (AD, BISH, MO, MPU, NY, P, PAP, PTBG, US, WU); slopes of Moouanui above Vaieenui Falls, on ridge top, below Mounanui, 2300 ft, 26 July 1988, Perlman & Florence 10174 (BISH, PTBG, US); Mounanui, on SW side of peak, in gulch back, at base of waterfall, 677 m, 9 September 1995, Perlman 14969 (AD, BISH, MO, P, PAP, PTBG, US, WU); Mt. Touaouoho, on NW side of peak, along ridges between Touaouho and Teavapuhiau, 616 m, 8 September 1995, Perlman & Wood 14964 (AD, BISH, MO, P, PAP, PTBG, US, WU), 725 m, 14962, (PTBG), 793 m, 14961 (PTBG, US), 2200 ft, 14965 (PTBG, US); Teavapuhiau Pass (above Ouia Valley), 700 m, 1–3 August 1977, Gagné 1245 (BISH); slopes and summit from Punaitai to Tekou summit, 830–1120 m, 25 July 1988, Wagner et al. 6193 (BISH, US), 850 m, 6201 (BISH, US).

##### Discussion.

When Fosberg and Sachet (1981) described the three varieties of *Cyrtandra ootensis* they stated that the species was not uniform in the variation of density of pubescence, density of toothing on leaf margins, length of petiole, in the length and openness of the inflorescence, and in the width of the corolla. They also pointed out that there was not much correlation among these variable characters, but nevertheless subdivided *Cyrtandra ootensis* into four varieties. The significant amount of new collections since those available to Fosberg and Sachet since 1981 have shown that there is essentially complete intergradations among these characters and there are no clear ecologically or geographically based morphological patterns, and we have therefore placed these names into synonymy. The only pattern of variation noted is the large-leaved more glabrate plants discussed under *Cyrtandra feaniana* that may represent hybridization on Fatu Hiva. Another case of possible hybridization on Tahuata is noted by the collections *Wood 6571*, which is included in *Cyrtandra ootensis*, butis intermediate toward *Cyrtandra feaniana*. *Wood 6570* collected close by is typical of *Cyrtandra feaniana*.

**Figure 10.**
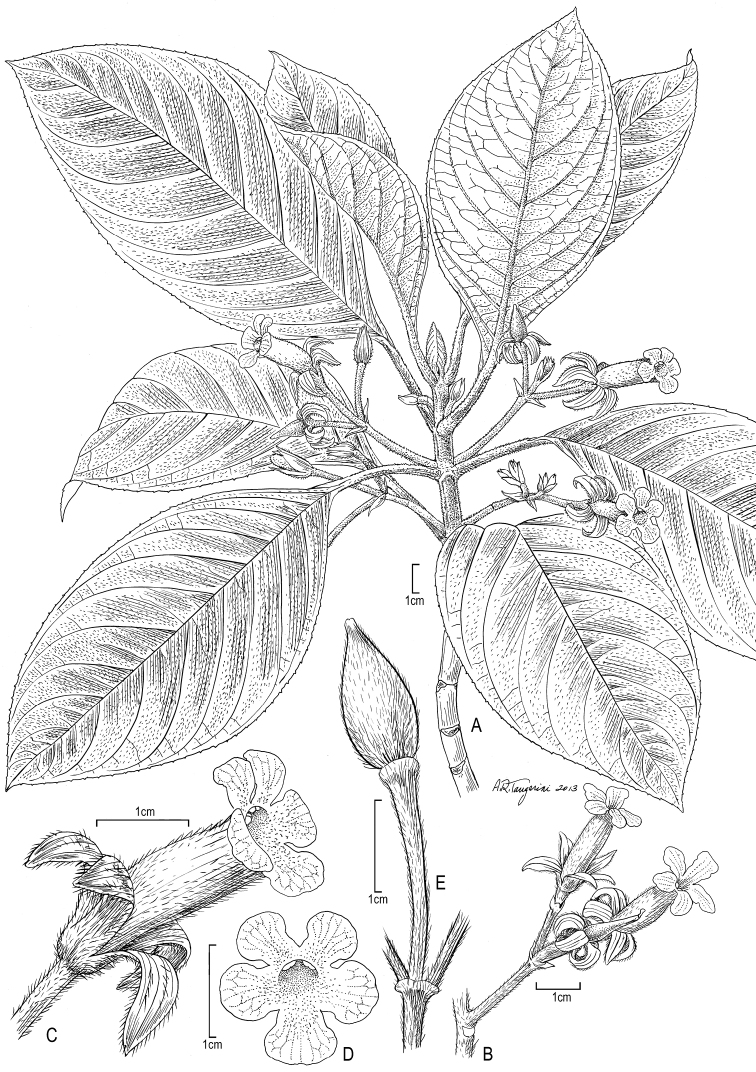
*Cyrtandra ootensis* F. Br. **A** Habit **B** Inflorescence **C** Flower, lateral view **D** Flower, face view **E** Fruit. Drawn from Perlman 19768 (US), except B from Wood 10235 (PTBG) and A also from Wagner 6193 (US) **B, C, D** augmented with photographs of Wood 10235.

#### 9. Cyrtandra tahuatensis

Fosberg & Sachet, Smithsonian Contr. Bot. 47: 31. 1981.

http://species-id.net/wiki/Cyrtandra_tahuatensis

Fig. 11

##### Type.

Marquesas Islands: Tahuata: Above Hamatea, on central crest of U’ua’o, 850 m, 31 May 1975, *J.-C. Thibault 83* (holotype: US-2969232! & US-2969234!, mounted on 2 sheets; isotype: US!).

##### Description.

Shrub to 1–4 m tall; stems ferruginous pubescent. Leaves opposite, densely ferruginous pubescent, elliptic to broadly elliptic, 12–22 × 5–9.5 cm, apex acuminate, base attenuate, margins serrate, petioles 2–5 cm long. Flowers 1–3 in cymes to 20 cm, peduncles 6–8.5 cm long, ca. 2–3 mm in diameter, pedicels 20–35 mm, bracts ca. 4 mm; calyx green, 12–20 mm, divided almost to base, densely ferruginous pubescent deciduous; corolla ca. 25 mm long, tube cylindrical, 18–20 mm long, lobes suborbicular; ovary ca. 8–14 mm, densely pilose, style 4–5 mm, densely pilose. Immature fruit narrowly ovoid, ca. 15 mm long, densely pubescent.

##### Distribution.

Marquesas Islands, occurring on Hiva Oa and Tahuata, from 690 to 830 m.

##### Ecology.

*Cyrtandra tahuatensis* is known from ridges and summit areas in wet forest and shrublandwith shrubs and trees such as species of *Coprosma*, *Crossostylis*, *Freycinetia*, *Ilex*, *Melicope*, *Metrosideros*, *Polyscias*, and *Weinmannia*.

##### Conservation status.

IUCN Red List Category: Endangered EN B1ab(i,ii,iii) + 2ab(i,ii,iii). B2: total area of occupancy less than 5000 km^2^ (ca. 376 km^2^). B1a, severely fragmented; B1b (i–iii), habitat continuing decline inferred. The suitable habitat for *Cyrtandra tahuatensis* on Hiva Oa (ca. 315 km^2^), and Tahuata (ca. 61 km^2^) is restricted to mountain slopes and summits, indicated as an endangered environment that is threatened by human activity (deforestation and fire), feral animals, and invasive plants, reducing the extent of the forest (Florence and Lorence 1997; Mueller-Dombois and Fosberg 1998; Meyer and Salvat 2009).

##### Specimens examined.

**Marquesas Islands. Hiva Oa:** Mt. Ootua, 750 m, 27 February 1975, Oliver & Schäfer 3223 (BISH, US); road from Atuona to Puamau, just below Mt. Ootua, 660-690 m, 22 January 1975, Sachet, Oliver & Schäfer 2127 (BISH, CBG, CHR, NSW, PTBG, US). **Tahuata:** ridge from Amatea to Moteve passing Meikaea, view down is on village of Hanatetena, on W side of ridge, 823 m, 13 July 1997, Perlman, Wood & Luce 15976 (MO, NY, P, PAP, PTBG, US, WU); ridge E of trail ridge up to Amatea from Kuaee, W facing slope, 774 m, 9°56'S, 139°4'W, 18 July 1997, Perlman, Wood & Luce 16010 (BISH, F, HAST, K, MO, NY, P, PAP, PTBG, US, WU); summit ridge near Haaiputeomo, satellite dish region NE of Vaitahu, 762-823 m, 9°57'19"S, 139°5'7.4"W, 17-19 July 1997, Wood 6553 (PTBG), Wood 6563 (AD, BISH, HAST, MO, NY, P, PAP, PTBG, US, WU); Amatea region, locations around Haaoiputeomo satelite dish (parabowl), 823 m, 9°9.2'S, 139°8'W, 4 Jul 2003, Wood 10266 (PAP, PTBG, US). **Fatu Hiva:** Mounanui slopes, from Teavapuhiau pass, in gulches below mt., 680 m, 10°28'49.7"S, 138°38'17.0"W, 6 Feb 2003, Perlman 18399 (BISH, P, PAP, PTBG, US); slopes of Mounanui, 719 m, 10°28'6.63"S, 138°38'18"W, 16 July 2005, Perlman 19667 (BISH, P, PAP, PTBG, US); Crete du Mt. Mounanui, 805 m, 10°28'S, 138°37'W, 26 July 1988, Florence & Perlman 9589 (PTBG, US).

##### Discussion.

*Perlman 18399, Wood 6563*, and *Wood 10266* were identified as *Cyrtandra ootensis* var. *mollissima* in the molecular study of Clark et al. (2009), but are here considered to represent *Cyrtandra tahuatensis*.

**Figure 11.**
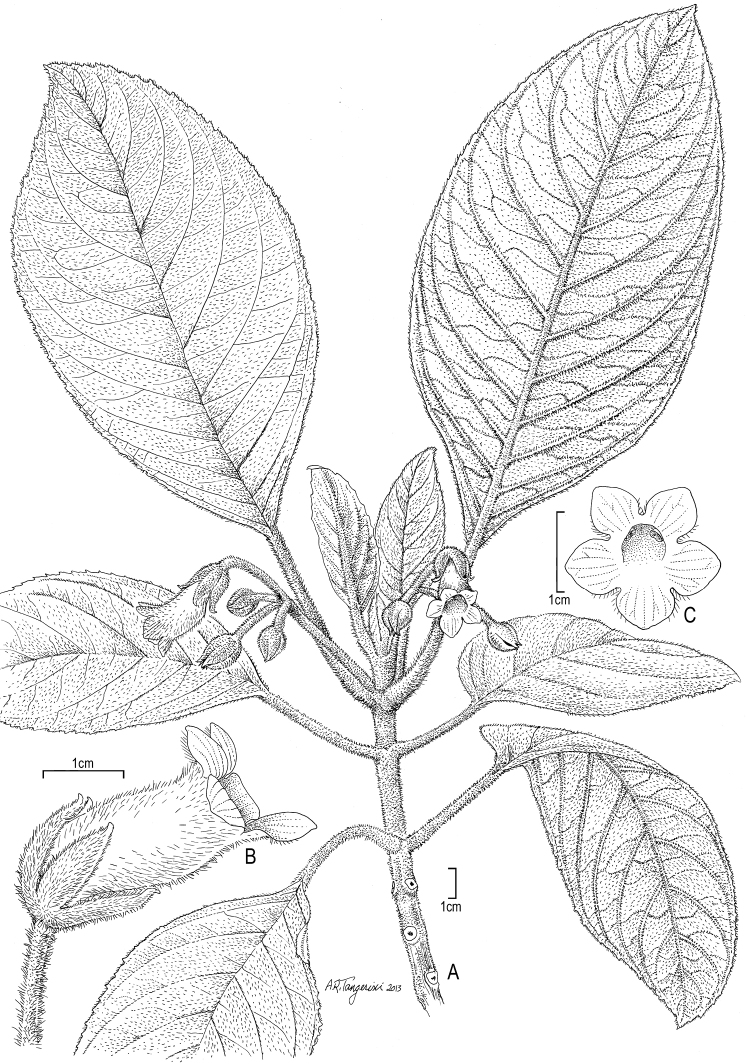
*Cyrtandra tahuatensis* Fosberg & Sachet **A** Habit, Wood 6563 (US) **B** Flower, lateral view, Perlman 18399 (US, PTBG) and photographs by Wood from greenhouse specimens grown at NTBG **C** Flower, face view, Perlman 18399 (US, PTBG) and photographs by Wood from greenhouse specimens at NTBG.

#### 10. Cyrtandra revoluta

Fosberg & Sachet, Smithsonian Contr. Bot. 47: 31. 1981.

http://species-id.net/wiki/Cyrtandra_revoluta

##### Type.

Marquesas Islands: Fatu Hiva: Base of Mt. Natahu, on cliff face, 828 m, 1–3 August 1977, B. H. Gagné (S. L. Montgomery coll.) 1276 (holotype: BISH-510504!; isotype: US!).

##### Description.

Shrub ca. 0.2–0.3 m, with thick fleshy stems, densely woolly pubescent. Leaves opposite, very crowded on upper 2–3 nodes, stiff-coriaceous, elliptic, 7–10 × 3–5 cm, margins strongly revolute, apex obtuse, bases attenuate to cuneate, upper surface thinly long pilose, glabrate, lower surface densely ferruginous pubescent, petioles 0.5–1 cm. Flowers in condensed cymes, 2–2.5 cm, crowded between the leaves, bracts elliptic, ca. 10–15 mm long, peduncles 5–8 mm, ca. 1–2 mm in diameter, pedicels 6–13 mm; calyx ca. 13–15 mm long, divided ca. 3/4 its length; corolla white (none available on specimen). Berry unknown.

##### Distribution.

In the Marquesas known only from the type collections on Fatu Hiva at 830 m.

##### Ecology.

The type was collected on a cliff face, but the specific ecology of *Cyrtandra revoluta* is unknown.

##### Conservation status.

IUCN Red List Category: Critically Endangered CR B2a + 2b (i, ii, iii). B1, total extent of occurrence less than 100 km^2^ (ca. 85 km^2^), a,b, known from a single location; B2a, estimated area of occupancy estimated to be less than 10 km^2^ [one collection known]; B2b (i–iii), habitat continuing decline inferred. The estimated area of occupancy for *Cyrtandra revoluta* on Fatu Hiva (less than 10 km^2^) is indicated as an endangered environment, threatened by human activity (deforestation and fire), feral animals, and invasive plant species, reducing the extent of the forest (Florence and Lorence 1997; Mueller-Dombois and Fosberg 1998; Meyer and Salvat 2009).

##### Discussion.

*Cyrtandra revoluta* is quite distinctive in its short stature and its stiff-coriaceous, revolute leaves. It is known from only one incomplete specimen making assessment of its relationships difficult. The deeply divided, green calyx suggests that it is part of the divided calyx group.

#### 11. Cyrtandra toviana

F. Br., Bernice P. Bishop Mus. Bull. 130: 271. 1935.

http://species-id.net/wiki/Cyrtandra_toviana

Fig. 12

##### Type.

Marquesas Islands: Nuku Hiva: Tovii, 800 m, October 1922, E. H. Quayle 1279 (holotype: BISH-509964!).

##### Description.

Shrubs 1.5–2 m tall; stems usually few. Leaves opposite, borne on upper few nodes, peltate, suborbicular, 7–10 × 6–9 cm, ferruginous glandular pubescent, margins coarsely dentate, petioles 5–10 cm. Cymes few-flowered, ca. 2.5–3 cm; bracts ca. 5–6 mm, deciduous, peduncles ca. 10 mm, ca. 1.5–2 mm in diameter, pedicels 12–25 mm; calyx campanulate, 10–17 mm, densely ferruginous glandular pubescent, divided ca. ¼–1/3 of length, lobes acute, 4–5 mm; corolla funnelform, tube ca. 25–35 mm, the lobes rounded, ca. 6–10 mm; ovary conical-ovoid, 5 mm, glabrous; style ca. 13 mm long, glabrous in the lower portion, slightly pubescent near the apex. Berry ovoid, ca. 15 mm long.

##### Distribution.

Marquesas Islands, very rare or perhaps extinct, endemic to Toovii Plateau, Nuku Hiva, ca. 800–900 m. It is known from three collections collected in 1844, 1922, and the most recent in 1982.

##### Ecology.

*Cyrtandra toviana* is known only in *Metrosideros collina* woodland.

##### Conservation status.

IUCN Red List Categories: Critically Endangered CR B2a + 2b (i, ii, iii). B1, extent of occurrence estimated to be less than 100 km^2^; B2, area of occupancy estimated to be less than 10 km^2^ (ca. 9 km^2^), and B2a, a single population known; b (i–iii), habitat continuing decline inferred. The suitable habitat for *Cyrtandra toviana* on Nuku Hiva (ca. 340 km^2^) is indicated as an endangered environment, threatened by human activity (deforestation), feral animals, and invasive plants, reducing the extent of the forest (Florence and Lorence 1997; Mueller-Dombois and Fosberg 1998; Meyer and Salvat 2009).

##### Specimens examined.

**Marquesas Islands. Nuku Hiva:** 1844, Le Bâstard (P [2]); Toovii, Quayle 1279 (BISH); Toovii, épaulement au-dessus du réservoir, 895 m, 8°52'S, 140°9'W, 7 December 1982, Florence 4324 (BISH, P).

##### Discussion.

*Cyrtandra toviana* is unique within Marquesan *Cyrtandra* with its peltate leaves and small campanulate calyx. In fact, it could well represent a separate introduction of the genus to the Marquesas Islands.

**Figure 12.**
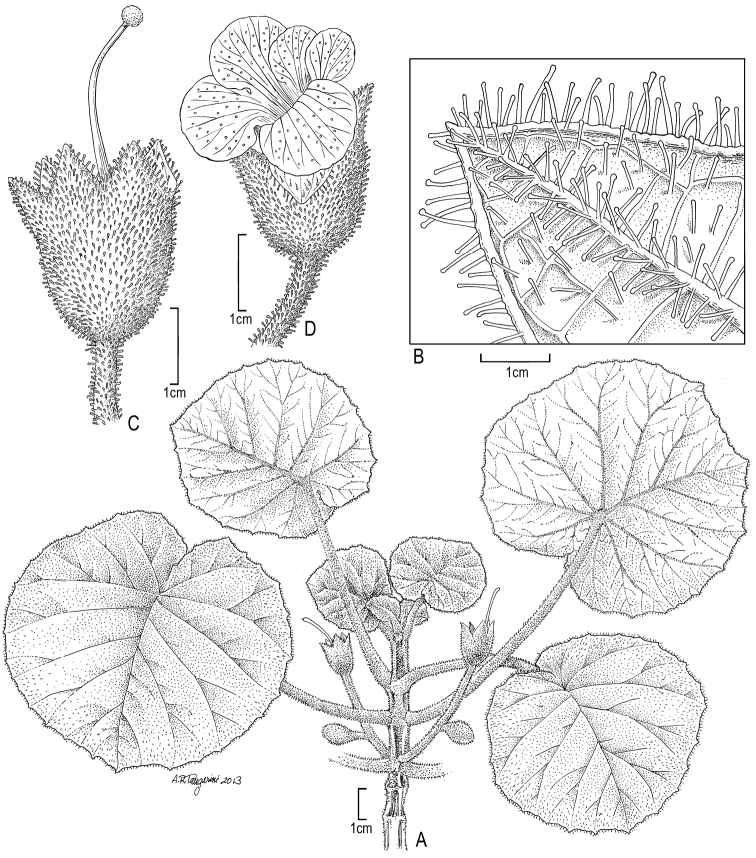
*Cyrtandra toviana* F. Br. **A** Habit **B** Leaf apex abaxial view **C** Calyx with pistil **D** Flower. Drawn from Florence 4324 (BISH).

### Putative hybrids

#### *Cyrtandra feaniana* × *Cyrtandra tahuatensis*

**Tahuata:** au-dessus de Hamatea, sur la crête centrale de U’ua’o, 850 m, 31 May 1975, Thibault 79 (US).

#### *Cyrtandra uapouensis* × *Cyrtandra kenwoodii*

**Ua Pou:** central Ua Pou including the summit crest regions around Oave and the near-by peak of Matahenua, 2950–3030 ft [899–924 m], 9°23'45.4"S, 140°4'43.3"W, 2–4 July 2004, Wood & Perlman 10830 (PTBG); ridge just north of Oave, between Oave and Matahenua, high mountain peaks along main backbone ridge, 945 m, 9°23'45.5"S, 140°4'43.3"W, 3 July 2004, Perlman & Wood 19082 (BISH, P, PAP, PTBG, US); Teavahaakiti, steep slopes of main ridge to S of Oave, N & E facing cliffs between Teavahaakiti & Tekohepu, 869 m, 5 July 1997, Perlman & Wood 15923 (PAP, PTBG, US, WU); Tekoheupu, summit ridge, 762–914 m, 9°24'31"S, 140°4'21"W, 4–5 July 1997, Wood & Perlman 6477 (PAP, PTBG, US).

## Supplementary Material

XML Treatment for Cyrtandra jonesii

XML Treatment for Cyrtandra nukuhivensis

XML Treatment for Cyrtandra thibaultii

XML Treatment for Cyrtandra uahukaensis

XML Treatment for Cyrtandra uapouensis

XML Treatment for Cyrtandra kenwoodii

XML Treatment for Cyrtandra feaniana

XML Treatment for Cyrtandra ootensis

XML Treatment for Cyrtandra tahuatensis

XML Treatment for Cyrtandra revoluta

XML Treatment for Cyrtandra toviana
